# Derivation, Characterization, and Stable Transfection of Induced Pluripotent Stem Cells from Fischer344 Rats

**DOI:** 10.1371/journal.pone.0027345

**Published:** 2011-11-04

**Authors:** Mikhail Liskovykh, Ilya Chuykin, Ashish Ranjan, Dina Safina, Elena Popova, Elena Tolkunova, Valentina Mosienko, Julia M. Minina, Natalia S. Zhdanova, John J. Mullins, Michael Bader, Natalia Alenina, Alexey Tomilin

**Affiliations:** 1 Institute of Cytology, Russian Academy of Sciences, St. Petersburg, Russia; 2 Max-Delbrück Center for Molecular Medicine, Berlin-Buch, Germany; 3 Institute of Cytology and Genetics, The Siberian Branch of the Russian Academy of Sciences, Novosibirsk, Russia; 4 The BHF/University Centre for Cardiovascular Science, Queen's Medical Research Institute, University of Edinburgh, Edinburgh, Scotland, United Kingdom; Brigham and Women's Hospital, United States of America

## Abstract

The rat represents an important animal model that, in many respects, is superior to the mouse for dissecting behavioral, cardiovascular and other physiological pathologies relevant to humans. Derivation of induced pluripotent stem cells from rats (riPS) opens the opportunity for gene targeting in specific rat strains, as well as for the development of new protocols for the treatment of different degenerative diseases. Here, we report an improved lentivirus-based hit-and-run riPS derivation protocol that makes use of small inhibitors of MEK and GSK3. We demonstrate that the excision of proviruses does not affect either the karyotype or the differentiation ability of these cells. We show that the established riPS cells are readily amenable to genetic manipulations such as stable electroporation. Finally, we propose a genetic tool for an improvement of riPS cell quality in culture. These data may prompt iPS cell-based gene targeting in rat as well as the development of iPS cell-based therapies using disease models established in this species.

## Introduction

For more than a century the rat has been an important animal model, which is superior in many respects to the mouse, for example for behavioral, cardiovascular and other physiological studies. Numerous inbred and outbred rat strains are used in different fields of research, and transgenic technologies are well developed in this species. However, until recently, gene targeting was not available in rats because ES cell derivation from pre-implantation rat embryos repeatedly failed [1], [2], [3]. This led to the mouse being used as the sole animal model for ES-cell based gene targeting techniques and for the establishment of tissue replacement therapies. A recent breakthrough finally allowed this problem to be overcome. It was shown that serum-free defined culture medium (N2B27) in conjunction with inhibition of the MEK (mitogen activated protein kinase)/ERK (extracellular signal regulated kinases 1 and 2) pathway and glycogen synthase kinase-3 (GSK3) by the small synthetic drugs PD0325901 and CHIR99021, respectively, in combination with activation of the LIF/STAT3 pathway (N2B27+2i+LIF), are required and sufficient to set and maintain the so-called “ground state” of pluripotent stem cells [4]. This empirical observation allowed ES cell line derivation from previously non-permissive mouse strains, such as NOD mice [5], [6] and ultimately, after 20 years of unsuccessful attempts, from rats [7], [8]. The established cell culture conditions were also demonstrated to be beneficial for the establishment and maintenance of pluripotent cells from various species including rat and human [4], [6], [7], [9], [10].

Induced pluripotent stem (iPS) cells are derived from somatic cells reprogrammed to the pluripotent state by the induced expression of defined transcription factors, achieved for the first time by the seminal work of Takahashi and Yamanaka [11]. This new type of pluripotent cells has offered new exciting options in regenerative medicine allowing the replacement of cells and organs with the patient's own cells thereby avoiding immunological complications. In order to develop such technologies in approved animal models, iPS cells were also generated from rodents. Despite numerous papers published describing mouse iPS cells, very few groups reported the derivation of iPS cells from rat (riPS). Interestingly, whereas inhibition of GSK3 and MEK/ERK pathway was found to be critical for the survival and maintenance of pluripotency in riPS cells in some studies [8], [12], [13], [14], the derivation of riPS cells using classical serum- and LIF-containing mouse ES medium was also reported [15].

In this study we applied the four reprogramming factors [11] to derive iPS cells from rat embryonic fibroblasts (REF) using different cell culture conditions. We report an improved protocol for the generation and maintenance of these cells using small inhibitors of the MEK/ERK pathway and GSK3. In addition, we present a method suitable for their genetic modification by stable transfection and propose a genetic tool for an improvement of riPS cell quality in culture.

## Materials and Methods

### Ethics statement

All animal procedures were performed according to the guidelines for the humane use of laboratory animals, with standards corresponding to those prescribed by the American Physiological Society. The teratoma formation and riPS cell injection into rat preimplantation embryos with subsequent analysis of chimeric embryos were performed in the Institute of Cytology strictly in agreement with the animal protection legislation acts of the Russian Federation and was approved as humane use of laboratory animals by the Institute's Ethical Board. The isolation of rat embryonic fibroblasts (REF) was performed using naturally mated animals which were sacrificed using a UK Home Office 'Schedule 1′ procedure which does not require specific ethical approval.

### Plasmids and lentiviruses

To induce pluripotency lentiviruses (human immunodeficiency virus (HIV)-based retroviruses), encoding pluripotency factors *Oct4, Sox2, cMyc,* and *Klf4* were generated. To this end full-size cDNAs of corresponding genes were amplified by PCR from reverse-transcribed mouse ES cell polyA RNA and cloned in place of the *EGFP* gene (Figure S3A) within the lentiviral vector LVTHM provided by Didier Trono [16]. 293T cells were transfected with envelope-encoding *pMD2G* (5 mg), packaging *pCMV-dR8.74PAX2* (5 mg), and either *Oct4-, Sox2*-, *Klf4-,* or *cMyc*-encoding LVTHM-based plasmids (20 mg) by calcium-phosphate method. Lentiviruses in cell culture supernatant were collected and processed as described elsewhere [16].

*Thymidine kinase* (*TK*) gene was PCR amplified from *pKO-NTKV-1907* (Stratagene) and cloned into *p2A2Btk-luc* plasmid [17] at NcoI/XhoI sites, replacing the luc sequence. The *iresPuro-polyA* cassette was subsequently cloned at XhoI/SpeI sites just downstream of the *TK* gene of the resulting plasmid to obtain the *p2A2Btk-TKiresPuro* vector (Figure S3B).

### Cell culture

Unless specified, all cell culture products were from Invitrogen (Darmstadt, Germany). Mouse and rat embryonic fibroblasts (MEF and REF, respectively) were routinely maintained in high glucose DMEM supplemented with 10% FBS (Sigma-Aldrich, St. Louis, MO), 100 U/ml penicillin, 100 µg/ml streptomycin, 2 mM L-glutamine, 1x non-essential amino acids, 50 µM β-mercaptoethanol. MEFs were inactivated by cultivation in cell culture media supplemented with 10 µg/ml mitomycin C (Sigma-Aldrich) for 2 hr. In reprogramming experiments 3 different media were used. ES media: Knockout DMEM supplemented with 15% ES cell-qualified fetal bovine serum (Sigma), 100 U/ml penicillin, 100 µg/ml streptomycin, 2 mM L-glutamine, 1x non-essential amino acids, 50 µM β-mercaptoethanol. SR media: Knockout DMEM supplemented with 20% KnockOut serum replacement, 100 U/ml penicillin, 100 µg/ml streptomycin, 2 mM L-glutamine, 1x non-essential amino acids, 50 µM β-mercaptoethanol. N2B27 media: mixture 1∶1 of N2-media (DMEM/F12 supplemented with 1x N2, 100 U/ml penicillin, 100 µg/ml streptomycin, 0.005% BSA, 25 µM β-mercaptoethanol) and B27 media (NBM supplemented with 1x B27 (without RA), 100 U/ml penicillin, 100 µg/ml streptomycin, 2 mM L-glutamine, 25 µM β-mercaptoethanol). When indicated, media were supplemented with 500 U/ml LIF (PAA, Pasching, Austria), 3 µM GSK3 inhibitor CHIR99021 (Axon, Groningen, Nitherlands), 1 µM MEK inhibitor PD0325901 (Axon), 0.5 µM A-83-01 (Tocris Bioscience, Missouri, USA).

### Derivation of riPS cells

To isolate REFs naturally mated Fischer344 female rats were checked for the presence of a vaginal plug and thereafter sacrificed at day 14 of pregnancy (E14). Isolated embryos were freed from head and visceral tissues, minced, subsequently trypsinized and plated on tissue culture dishes (passage 0). REFs at passage 3 were seeded at 1.5×10^4^ cells/cm^2^ in a 6-well plate and transduced 24 hrs later with the LVTHM-based Oct4, Sox2, Klf4, cMyc, and EGFP lentiviruses (see above). Four days later cells were split onto a 10 cm culture dish (Falcon), containing mitomycin-inactivated MEFs and cultured in one of the three reprogramming media (see above). After 10-12 days, riPS cell colonies were picked, dissociated in TrypLE-express, and expanded on MEF-feeder cells in the respective reprogramming medium.

### Maintenance and electroporation of riPS cells

riPS cells were routinely maintained on mitomycin-treated MEFs in N2B27+2i+LIF media. Cells grew as round, compact colonies, which tended to detach after 2-3 days in culture. Cells were routinely replated every 3–5 days using TrypLE-express. Depending of the proportion of attached cells, riPS cells were either trypsinized on the plate, or collected from the supernatant, centrifuged, and dissociated in suspension. This procedure allowed removal of dead feeder cells as well as differentiated riPS cells, which were usually stably attached to the substrate. For stable transfection, riPS cells were harvested with TrypLE-express, and subsequently co-electroporated (5×10^6^ cells,^,^ 240 V, 500 µF) with 25 µg of circular *pMC-Cre* (kindly provided by K. Rajewsky) and linearized *p2A2B-tk-TKiresPuro* plasmids (7∶1 molar ratio). Puromycin (2 µg/ml) was added 48 hrs later for 3 days; clones were picked 10 days after electroporation into 96-well plates and genotyped by PCR, using primers specific for exogenous *Oct4, Klf4, Sox2, cMyc*, and *EGFP* and for the *2A2B-tk-TKiresPuro* cassette (primer sequences are listed in the Table S3). Three subclones, derivatives of the clone IIIB9 were chosen for further analysis. riPS cells harboring the *2A2Btk-TKiresPuro* cassette were occasionally cultured in the presence of 1 µg/ml puromycin (usually for 3 days every 4–5 passages).

### riPS cell karyotyping

Six primary male riPS cell clones, selected by Y-chromosome-specific PCR of genomic DNA, and three sub-clones after Cre-mediated lentivirus excision, were subjected to karyotyping procedure as follows. Exponentially growing riPS cells were incubated for 1 hr with 5 µg/ml ethidium bromide, then for 1.5 hrs with 0.5 µg/ml colchicine. Cells were next harvested with TrypLE and incubated for 20 min at 37°C in 0.075 M KCl solution, fixed in methanol/acetic acid (3∶1), placed on pre-wet chilled microscope slides, air-dried, stained for 5 min either with Giemsa stain or with 200 ng/ml 4′,6-diamidino-2-phenylindole (DAPI) in 2xSSC buffer. Slides were rinsed with 2xSSC and distilled water, then embedded under coverslips into antifading solution (Vector). DAPI-stained chromosomes were identified as described elsewhere [18]. Giemsa-stained metaphase spreads (90–140) were used for chromosome counts and polyploid cells determination. To identify chromosomes from 9 to 66 DAPI stained metaphase plates were analyzed. From 9 to 40 metaphase spreads were examined in clones containing from 30 to 50 percent of polyploid cells. DAPI-stained metaphase spreads were analyzed using the AxioPlan 2 Imaging microscope (Zeiss, Germany) equipped with CCD camera (CV M300, JAI Corporation, Japan), CHROMA filter sets and ISIS4 image-processing package of MetaSysteme GmbH at the Center for Joint Ownership for Microscopic Analysis SB RAS (ICG SB RAS, Novosibirsk).

### Immunocytochemistry and alkaline phosphatase staining

Cells were fixed in 4% paraformaldehyde (Sigma) and stained with antibodies against Oct4 (sc-5279, Santa Cruz Biotechnology Inc), SSEA-1 (MC-480, Developmental Studies Iowa Hybridoma Bank), Nanog (REC-RCAB0002P-F, COSMO BIO CO., Tokyo, Japan), and neuronal class III β-Tubulin (TUJ1) (MMS-435P, Covance), as described elsewhere [19]. Immunostained cells were examined either on a fluorescent microscope Leica DM6000 with 5x, 10x, 20x, and 40x air objectives or a confocal Leica TCS SP5 microscope with 20× air and 63× oil immersion objectives. UV (405 nm), Argon (488 nm) and HeNe (633 nm) lasers were used to excite the fluorophores. Images were acquired using the Leica TCS SP5 software. For the alkaline phosphatase (AP) staining, cells were fixed with 4% paraformaldehyde, washed with Tris-maleate buffer pH 9.0, and subsequently incubated for 30 minutes in Tris-maleate buffer, 4 mM MgCl_2_, 0.4 mg/ml N-AS-MX (Sigma), 1 mg/ml Fast Red TR Salt (Sigma). Once red colonies were detected the reaction was stopped by removing colored solution and adding PBS, pH 7.4.

### Clonability assay

To evaluate the effect of different small molecules on maintaining the self-renewal riPS cells were trypsinized into single cells and seeded at the density of 50 cells/cm^2^ in a 6-well plate on mitomycin-treated MEFs. Cells were cultured for 5 days in N2B27 media containing different combinations of inhibitors and subsequently fixed and subjected to the AP staining. Each condition was done in triplicate. The number of AP-positive colonies for each condition were averaged from 3 wells to evaluate the percentage of undifferentiated clones. To assess the viability the total number of survived colonies per well was counted.

### *In vitro* differentiation of riPS cells

The *in vitro* differentiation of riPS cells was carried out by the “hanging drop” method. riPS cells were dissociated by TrypLE express, resuspended in N2B27 medium containing CHIR99021, and plated in hanging drops (800 cells per 20 µl drop). Embryoid bodies, formed in hanging drops were collected after 2 days in 10 ml of N2B27 (w/o CHIR99021) and additionally cultured for four more days in low-adhesive bacterial Petri dishes. Medium was changed every second day. After 6 days, embryoid bodies were harvested and transferred onto Matrigel-coated dishes, approximately 10–12 embryoid bodies per 1-well of a 6-well plate in N2B27 medium. 6–8 days later differentiated cells were either fixed in 4% paraformaldehyde in PBS and analyzed further by immunocytochemistry or used for the RNA extraction and subsequent RT-PCR analysis.

### Teratoma formation

riPS cell subclones H5 and G3, exponentially growing on mitomycin-treated feeder layer in N2B27+2i+LIF medium, were treated for 3 days with puromycin (1 µg/µl), harvested with TrypLE express, resuspended in PBS and injected subcutaneously into athymic RNU rats (5−10×10^6^ cells) or CD-1 NUDE mice (1−2×10^6^). After 6–8 weeks teratomas were removed from euthanized animals and processed for histological analysis.

### RiPS cell injection into rat preimplantation embryos

riPS cells (subclone H5) were trypsinized, resuspended in a small amount of N2B27 medium and kept on ice until the injection. Embryos were recovered by flushing the excised oviducts and uterine horns with M2 medium (Sigma) from 2–5 months old naturally mated female Sprague-Dawley-Hannover (SD) outbred rats (Janvier, France) at day 4 post coitum, as previously described [20], [21]. 5–7 riPS cells were injected into 8 cell-stage embryos placed in a drop of M2-medium under mineral oil. Immediately after microinjection embryos were transferred into uterine horns of anesthetized pseudopregnant (day 4 post coitum) SD females (10–12 embryos per recipient) [20]. To examine the development of fetuses and to recover live pups the recipient rats were euthanized on day 18 of gestation. DNA was isolated for PCR analysis from multiple embryonic tissues, including the tail, liver, kidney, heart, and head, and also from extraembryonic tissue (placenta). Subclone H5, used in this approach, harbors the *2A2Btk-TKiresPuro* cassette in its genome. A primer pair specific for this cassette was used to distinguish between incorporated H5 riPS cells and host embryo cells (primer sequences are listed in the Table S3). The same primer pair and primers specific for the rat *angiotensinogen (AOGEN)* gene (as an endogenous control) were used for the evaluation of % chimerism in E18 fetuses by real time PCR (see below). Standard curve was calculated by mixing H5 cells (containing *2A2Btk-TKiresPuro* cassette) and genetically unmodified rat cells (WT) at different ratios: H5 only, H5∶WT 1∶4, 2∶3, 3∶2, 1∶4, WT alone. H5 alone was taken as 100%. Real time PCR was performed in technical duplicate.

### Fluorescence-activated cell sorting (FACS) and real-time PCR

For cytometric analysis 10^7^–10^8^ riPS IIIB9 cells (p22–p24) were harvested with TrypLE-express, washed with PBS, and subsequently resuspended in 100 µl of PBS. Probes were sorted by flow cytometry on FACSAria2 (BD Biosciences). 10^5^ GFP-positive and GFP-negative cells were plated to 6-well plates and GFP fluorescence was monitored over 5 days in culture. Cells were examined under a fluorescent microscope before plating to exclude the presence of green cells in the GFP-negative population. The rest of sorted cells were used for RNA preparation. For RT and real time PCR analysis RNAs were extracted by RNA mini kit (Qiagen). Residual genomic DNA was removed by DNase I treatment (Qiagen). RNA was reverse transcribed using random hexamers and Moloney murine leukemia virus reverse transcriptase (M-MLV, Invitrogen). The real-time PCR approach used the SYBR green reagent and a iQ5 BioRad cycler as described [19]. An established method was applied to compare gene expression levels between groups, using the equation 2^−ΔΔCT^ [22]. Gene expression was normalized to Nat1 mRNA expression. The primers used are listed in Table S3. Real time PCR was run in technical duplicate.

## Results and Discussion

To derive rat iPS (riPS) cells, rat embryonic fibroblasts (REFs) were transduced with *Oct4*, *Sox2*, *Klf4*, *cMyc* and, for visualization, with *EGFP*-encoding lentiviruses (OKSM+G) and then plated at a low density on mitomycin-inactivated mouse embryonic fibroblasts (MEF) feeder layer in media permissive for the propagation of iPS cells. In the first set of experiments we applied the original cell culture conditions, described for mouse iPS cell derivation [11] using serum- and LIF-containing medium. However, in contrast to previously reported success in riPS cell derivation using similar transduction and cell culture systems [15], this approach did not result in any viable riPS cell colonies (Table 1).

**Table 1:** Efficiencies of riPS cell derivation in different culture conditions.

	Reprogramming Medium	References	Reprogramming efficiency	Total N of picked clones	Total N of survived clones	Survival rate after 2 passages
1	DMEM + 15% FCS +LIF	[11], [15]	0	0		
2	DMEM + SR +2i + LIF + A-83-01	[14]	8.9±1.3×10^−4^	48	37	25.0±12.5%
3	N2B27 +2i + LIF	[7]	5.4± 0.9×10^−4^ *	48	12	70.8±6.3%*

To derive rat iPS (riPS) cells 1.5×10^5^ REFs were transduced with the LVTHM-based Oct4, Sox2, Klf4, cMyc, and EGFP lentiviruses. Four days later cells were split onto a 10 cm culture dish and cultured in one of the three reprogramming media. After 10–12 days, primary riPS cell colonies were counted to calculate the reprogramming efficiency. Survival rate was calculated as a relation between the number of picked and the number of surviving clones after 2 passages.

*p<0.05, Student's *t*-test condition 3 *vs* condition 2, [DMEM + SR +2i + LIF + A-83-01] N = 3; [N2B27+2i + LIF]: N = 4 independent experiments.

**Figure 1:**
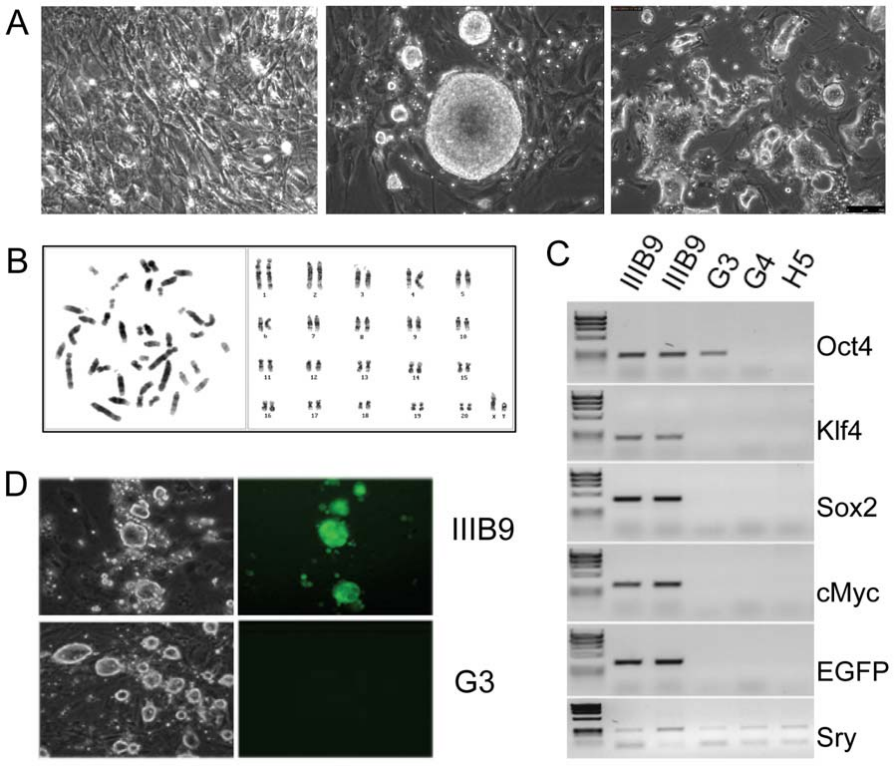
Generation of rat iPS (riPS) cells. (**A**) Rat embryonic fibroblasts (REFs) (left), primary riPS cell colony derived thereof after transduction with lentiviruses expressing *Oct4*, *Klf4*, *Sox2*, *cMyc*, and *EGFP* with some satellite colonies (middle), and riPS cell line after several passages in N2B27+LIF+2i medium (right). (**B**) Karyotyping results of the G4 riPS cell clone, in which 90% of metaphase plates showed the normal male chromosomal content (42,XY). (**C**) Cre-mediated excision of the proviruses in riPS cell clones H5, G3, and G4 from the genome of the parental IIIB9 riPS cell clone, shown by PCR of genomic DNA with primers specific for exogenous *Oct4*, *Klf4*, *Sox2*, *cMyc*, and *EGFP* sequences. PCR for the Y-chromosome specific gene Sry confirmed the DNA integrity and male origin of riPS clone IIIB9. (**D**) Cre-mediated excision of EGFP-lentivirus visualized in G3 riPS cell clone under fluorescent microscope.

Taking into account the recent success in the generation of rat ES cells [7], [8], we then applied the N2B27+2i+LIF culture conditions and achieved a robust derivation of riPS cells from REFs with the help of (OKSM+G) lentiviral vectors (Table 1, Figure 1A, Figure S3A). The inclusion of the cMyc virus, although beneficial for the efficiency of riPS cell derivation, was not obligatory (data not shown). We next compared the N2B27+2i+LIF with previously reported most efficient riPS cell-derivation medium containing serum replacement (SR), same MEK and GSK3 inhibitors (2i), LIF, and additionally, the activin receptor-like kinase (ALK5) inhibitor A-83-01 [14]. The numbers of primary riPS cell colonies were slightly higher in the SR-based medium, however, the percentage of surviving clones following 2 subsequent passages was 71% in the N2B27-based vs. 25% in the SR-based medium, respectively (Table 1).

Upon derivation eight individually picked clones, reprogrammed in N2B27+2i+LIF conditions were propagated to establish riPS cell lines. Cells were routinely maintained on inactivated MEFs in N2B27+2i+LIF medium. Rat iPS cells typically grew as compact clumps that tended to detach from substrate as colonies expanded, resembling in this respect rat ES cells [8]. However, after rounds of trypsinization and passaging, riPS cells changed their morphology, and flat and more adhesive colonies appeared. These features make riPS cells notably different from those of mouse origin. Also noticeable, primary riPS cell colonies were frequently surrounded by small satellite colonies (Figure 1A, central panel). With respect to these unusual morphological characteristics, it would be of interest to compare, for example, properties and expression levels of matrix adhesion molecules in iPS and ES cells of both species.

**Figure 2:**
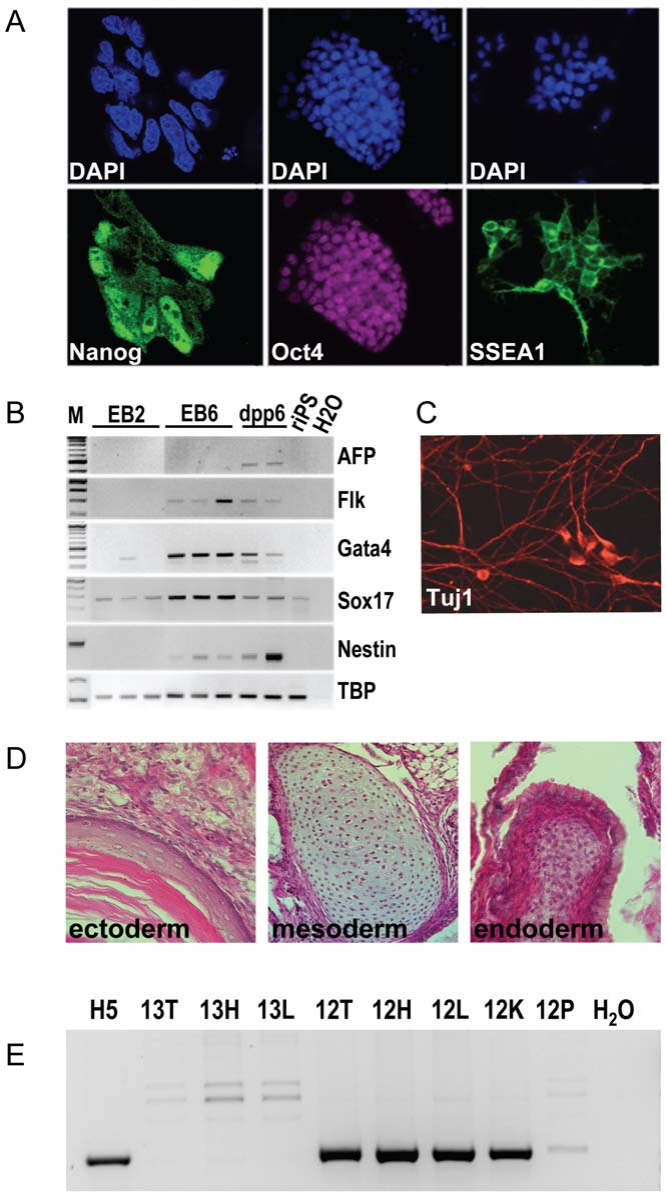
Pluripotency of generated riPS cells. (**A**) Immunostaining of cultured riPS cells for Nanog (left) (63x objective, confocal imaging, clone IVC2), Oct4 (middle, clone IIIB9), and SSEA1 (right, clone G4) (20x objective, fluorescent microscope). (**B**) RT-PCR analysis of gene expression in undifferentiated riPS cells (clone H5) and in cells during the course of *in vitro* differentiation via the embryoid body (EB) protocol after 2 days (EB2), 6 days (EB6) and 12 days (dpp6, 6 days post plating) of differentiation; shown is the analysis of mesodermal (*AFP, Flk1*), endodermal (*Gata4* and *Sox17*), and ectodermal marker (*Nestin*) expression. (**C**) Differentiation of riPS cells into neurons *in vitro*, shown by immunostaining with Tuj1 antibodies at dpp6 of differentiation (clone H5). (**D**) H&E staining of sections of teratomas formed after a subcutaneous injection of riPS cells (clone G3) into NUDE rats; shown are stratified epithelium (left), cartilage (middle), and epithelium, likely of endodermal origin (right), belonging to ectodermal, mesodermal, and endodermal germ layers, respectively. (**E**) riPS cells (clone H5) injected into 8-cell rat embryos can contribute to the formation of the embryo proper and only to a very limited extend to the placenta, as shown by PCR analysis of day 18 rat embryo DNA. H5 – riPS cell clone used for the injection, 12 and 13 – positive and negative fetuses, respectively. T – tail, L – liver, H – head, K – kidney, P – placenta.

Immunostaining for the pluripotency markers revealed that riPS cells are positive for Oct4, Nanog, SSEA1 and alkaline phosphatase (Figure 2A and Figure S4 A–C). Interestingly, despite the presence of inhibitors which were shown to have a beneficial effect on Nanog expression in mouse ES cells [23], in analysed riPS cell lines Nanog showed a variable expression pattern, consistent with previous observations in mouse ES cells grown in serum-containing medium [24]. Further analysis showed that riPS cells were able to form embryoid bodies and give rise to derivatives of all three germ layers in course of *in vitro* differentiation (Figure S4D). The clonability assay showed that riPS cell proliferation and survival were severely compromised in the absence of GSK3 inhibitor CHIR99021, whereas omission of PD0325901 and LIF from the culture medium led to the quick differentiation of riPS cells (Table S1; Figure S2). Such a tight dependence upon the two inhibitors and LIF during the derivation and maintenance indicates that the newly obtained riPS cells are in the ground state of pluripotency [4], [7].

Because male ES cells are strongly preferred for gene targeting in mouse, we reasoned that the same would apply to the rat iPS cells. Thus we determined gender of primary riPS cell clones by PCR with Sry-specific primers (Table S3 and data not shown). Six selected clones were further karyotyped as shown in Figure 1B and Table 2. All, except IIIA10, which is likely to be a mixed clone, were confirmed to be XY. A characteristic of all six clones was a high ratio of tetraploid and aneuploid cells (Table 2). The latter cells were predominantly hypoploid with different chromosomes being lost (data not shown). The clone that demonstrated the highest ratio of cells with normal karyotype (IIIB9) was used for subsequent manipulations. Of note, chromosomal instability appears to be a hallmark of cultured ES cells. A recent study reported an amplification of centrosomes, leading to both polyploidization and aneuploidization of human ES cells [25]. Extensive analysis of 540 mouse ES cell lines showed that only 60% possess a normal diploid karyotype and 25% show a loss of the X chromosome [26]. Significant chromosomal instability was also reported for mouse and human iPS cells [11], [27], [28]. The rate of chromosomal abnormalities may be a consequence of both culture conditions and intrinsic properties of pluripotent cell lines. It is also possible that chromosomal instability is a consequence of the dramatic change in epigenetic state of cells during reprogramming.

**Table 2:** Summary of karyotype analysis of established riPS cell lines.

riPS cell clone/subclone	Karyotytes in diploid cells	% of diploid metaphase	% of aneuploid metaphase	% of polyploidy metaphase
IVF3	42,XY	60	17	23
IVF2	42,XY	50	50	N/A
IVC2	42,XY	68	9	23
IIIA10	42,XY/42XX	45	27	28
IVG3	42,XY	25	25	50
IIIB9	42,XY	79	1	20
G3*	42,XY	58	36	6
G4*	42,XY	90	6	4
H5*	42,XY	63	58	8

*Subclones of IIIB9 following Cre-mediated lentivirus excision.

Although HIV-derived lentiviruses are considered to efficiently resist epigenetic silencing in the majority of cell types, this is clearly not the case with riPS cells. For example, we observed a high rate of EGFP-encoding lentivirus silencing in newly derived riPS cell clones, which became increasingly obvious with increasing number of passages (Figure S1A–C). The same phenomenon has also been observed in human iPS cells by others [14]. Interestingly, while the silencing appeared in undifferentiated cells, reactivation of lentiviral expression was observed in some spontaneously differentiated cells (Figure S1D, E). Reset of the expression of either *Oct4*, *Sox2*, or *Klf4* in a subset of riPS cells might have an adverse effect on their further differentiation to specific cell types, whereas reactivation of the *cMyc* provirus after its germline transmission has been reported to induce tumor growth [29], [30]. Therefore, we set out to delete the proviruses from the genome of the reprogrammed cells, making use of the loxP sites included in the proviral LTRs [16]. To this end, the riPS cell clone IIIB9, chosen on the basis of the highest ratio of cells with normal karyotype (Table 2), was co-electroporated with Cre recombinase-encoding *pMC-Cre* plasmid and linearized *p2A2Btk-TKiresPuro* plasmid. The latter, besides being useful for an enrichment of Cre-electroporated cells via a rapid puromycin selection, resulted in a dramatic improvement in the quality of riPS cell cultures (see below). Provirus excision was monitored by PCR with primers specific to exogenous (lentivirus-encoded) *Oct4*, *Sox2*, *Klf4*, *cMyc*, and *EGFP* sequences (Figure 1C, D and Table S3). Out of 96 analyzed riPS cell subclones two (G4 and H5) were found to have all 5 proviruses excised (Figure 1C, D, Table S2). The analyzed subclones showed modal 42,XY karyotype (Table 2 and Figure 1B) and were able to maintain iPS cell morphology and expression of the pluripotency markers, (Fig. 2A and data not shown). Secondly, the riPS cell subclones were able to differentiate *in vitro* by formation of embryoid bodies (Figure 2B, C). Analysis of *in vitro* differentiation ability showed that the procedure is much more demanding in comparison to mouse iPS cells. Either the use of serum-containing medium or the formation of aggregates from single cell suspension in non-adherent dishes in serum-free conditions both led to massive cell death at day 3–5 of differentiation (data not shown). The hanging drop method using serum-free medium supplemented with the GSK3 inhibitor CHIR99021 during the first 2 days of differentiation, similar to the approach reported for the rat ES cells [8], allowed us to address the riPS cell viability issue. Thirdly, when injected into the immune-compromised NUDE rats or mice, the riPS cell subclones formed teratomas consisting of cell types derived from all three germ layers (Figure 2D). Lastly, riPS cell subclones could efficiently participate in embryonic development following their injection into rat preimplantation embryos (Figure 2E). Out of 11 fetuses analyzed one showed a significant degree (∼20%) of chimerism (Table S4). Significant incorporation of differentiated riPS cell descendants into tail, head, liver and kidney was tracked by PCR of genomic DNA using primers specific for the *2A2Btk-TKiresPuro* cassette (Figure 2E, Figure S3B). In contrast, very limited contribution to the placenta was detected (Figure 2E), suggesting that riPS cells, like iPS and ES cells of other species, lack the ability to differentiate into the trophoblast cell lineage. Taken together, our data suggest that the newly derived cell lines represent *bona fide* iPS cells and that Cre recombinase-mediated excision does not produce any obvious chromosomal rearrangements or loss of pluripotency.

**Figure 3:**
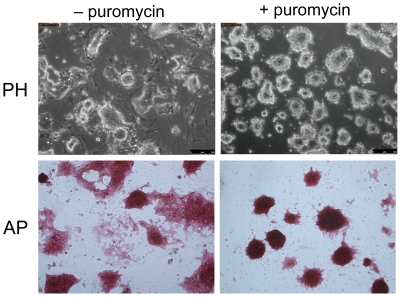
Positive selection of undifferentiated riPS cells *in vitro* . riPS cells (here clone H5) harboring the *2A2Btk-TKiresPuro* transgenic cassette show an increasing percentage of spontaneously differentiated cells with flat morphology and reduced alkaline phosphatase (AP) expression with increasing number of passages in N2B27+2i+LIF medium (left column), whereas the differentiated cells have been mostly eliminated from the culture following a 3-day selection in the same culture medium containing 1 µg/ml puromycin (right column). PH – phase contrast.

Despite of the presence of LIF and both small inhibitors, riPS cells showed some predisposition to spontaneous differentiation *in vitro* and could be maintained in an undifferentiated state without subcloning only for a limited number of passages. To address this issue, we have developed a transgеnic cassette that allowed a selective and efficient elimination of differentiated cells in riPS cell cultures. The 200-bp *2A2B* sequence from the distal enhancer of *Pou5f1* (*Oct4*) gene, containing binding sites for Oct4 and Sox2 heterodimers (*2B*) and a DNA binding element of an unknown transcription factor (*2A*), has been shown to act synergistically in transient transfection as an ES cell-specific enhancer when coupled to either the homologous *Pou5f1* or to a heterologous minimal promoter such as thymidine kinase (*tk*) [17]. Our data suggests that the *2A2Btk* enhancer/promoter moiety can direct pluripotency-specific expression of transgenes not only transiently, but also when stably delivered to the genome of ES and iPS cells (data not shown). Making use of this property, we placed a two-way selection bicistronic cassette (*TKiresPuro*) under transcriptional control of *2A2Btk* (Figure S3B) and stably delivered the resulting construct into the genome of riPS cells via electroporation. This manipulation, performed in parallel with the transient delivery of Cre recombinase, allowed us to reliably improve, through brief courses of puromycin selection, the quality of riPS cell cultures via an efficient elimination of spontaneously arising differentiated cells (Figure 3). Of note, we found this cassette functioning equally well in mouse iPS cells, as well as in ES cells of both species (data not shown). The cassette also facilitated enrichment for differentiated cells *in vitro* (via the *TK* gene, data not shown). To our knowledge, the *2A2Btk* moiety represents the shortest known regulatory element sensitive to the differentiation status of ES and iPS cells. In addition, the relatively small size of this cassette allows stable genome integration via lentiviral vectors (data not shown).

We have reported in this paper an optimized approach for the derivation of riPS cells which makes use of various previously reported improvements of iPS/ES-related methods. While comparable in term of derivation efficiency, our approach offers a better survivability of picked riPS cell clones after subsequent culturing, compared to the previously published non-excisable lentivirus-based serum-free system [14]. Contrary to yet another report [15], we failed to generate any viable riPS cells using serum-containing culture medium, even with serum, which was well permissive, for instance, for mouse iPS cell derivation (data not shown). Thus our data emphasize the importance of using serum-free medium for consistent results with riPS cell derivation. During the time course of our studies another group reported a riPS cell derivation protocol that used both REFs and rat neural precursors as starting cell types and relied on serum-containing medium, non-excisable moloney murine leukemia virus (MMLV)-based delivery (in combination with ES cell extracts), and mitotically inactivated REFs as feeder cell layer [13]. A direct comparison of this and our method is not feasible. However, our approach seems to offer several advantages: (1) well-known superior reprogramming capacity of HIV-based, compared to MMLV-based retroviral vectors; (2) a true hit-and-run strategy involving lentivirus excision from the rat genome (without any obvious chromosomal rearrangements) and, thus, the elimination of the risks associated with reprogramming factor re-activation and, as a subsequence, altered differentiation properties of riPS cells; (3) the approach as it has been discussed above, eliminates the dependence on serum batch variation. Recently proposed virus-free iPS derivation systems [31], [32], [33], [34], [35] remain to be tested in the rat, however, for the time being, the hit-and-run protocol offered in this paper appears to be the most efficient and consistent.

Despite rapid progress in the development of novel genomic technologies, such as RNA interference [36] and zinc-finger nuclease technologies [37], gene targeting via homologous recombination in ES cells currently remains the most straightforward approach in functional genomics. This approach, however, has not been available for rat until recently, when rat ES cells have been established [7], [8], and subsequently shown to be amenable to homologous recombination [10], [38]. In this regard, riPS cells may represent a viable alternative to rat ES cells for functional genetics because their derivation, as shown in this and other studies, is rather simple and straightforward and does not require a supply of preimplantation embryos which can be a quite limited for some strains of rat [[39] and E.P., unpublished data], relying instead on easily preserved stocks of rat embryonic fibroblasts. In particular, riPS cells can be isolated from the plethora of existing inbred rat models for polygenic diseases [40], such as hypertension, diabetes or epilepsy, and used to elucidate the pathogenetic mechanisms involved in these disorders. Compared to ES cells, riPS cells are preferable for the development of tissue replacement therapies, allowing the isolation of recipient-specific pluripotent cells and overcoming the problem of post-transplantation rejection. Lastly, due to the fact that, to date, models of many human degenerative diseases have been established solely in the rat, riPS cells represent a very attractive tool for the validation of therapeutical applications of iPS cell technologies in preclinical studies.

## Supporting Information

Figure S1 **Lentivirus silencing in undifferentiated riPS cells.** **(A)** riPS cell clone IIIB9 after 22 passages (p22) in culture. Notice round compact, morphologically undifferentiated colonies that have lost EGFP expression. (**B**) FACS for EGFP^+^ cells of riPS cell clone IIIB9 p22 (lower panel) and its subclone IIIB9-G3 derived following the excision of proviruses, including *EGPF*. The latter served as a negative control (upper panel). Similar results were obtained in 2 independent experiments. **(C)** Real time PCR analysis of sorted cells, showing that EGFP^—^ riPS cells express high levels of Nanog, confirming the pluripotent state of this cell population. IIIB9: N = 3; EGFP^—^ , EGFP^+^: N = 2. (**D**) The EGFP^—^ fraction of riPS cells after FACS shows the onset of EGFP expression in some cells already after 1 day in culture. **(E)** EGFP^—^ fraction cultured for further 4 days shows a substantial number of EGFP^+^ cells. Note, that most of Oct4^+^ cells do not express EGFP, whereas some of morphologically differentiated cells became green.
(DOC)

Figure S2 **RiPS cell clonability assay.** Representative images of alkaline phosphatase staining of riPS cells, seeded at a density of 500 cells per well of a 6-well plate and cultured for 5 days in the presence of LIF and 2i (A, B), or in the presence of LIF and CHIR99021 (C, D). 2.5x magnification (A, C). Examples of an undifferentiated colony (B) and of a morphologically differentiated colony with weak AP-staining (D) (20x and 10x magnification, respectively). Similar results were obtained in 2 independent experiments.
(DOC)

Figure S3 **Plasmids and lentiviruses.** (A) Map of the lentiviral vectors used in this study. Oct4, Sox2, Klf4, and cMyc cDNAs were cloned in place of the EGFP cDNA of the LVTHM vector, which is described in greater details in elsewhere (Wiznerowicz and Trono, 2003, *J. Virol* 77:8957-61). During reverse transcription, the U3 region of the 5′ LTR is synthesized by using its 3′ homologue as a template, which results in a duplication of LoxP site in the provirus integrated in the genome of transduced cells. The part of LV DNA between the loxP sites is subject to Cre-mediated excision. LTR, cPPT, and WPRE are lentiviral elements required for its integration and expression. (B) Scheme of the *2A2Btk-TKiresPuro* cassette (see text for abbreviations). Arrows indicate primers which were used to detect the cassette in the riPS cell transfected clones. The same primer pair was employed for the evaluation of % chimerism after injection of riPS H5 cells into rat preimplantation embryos (Figure 2E and Table S4).
(DOC)

Figure S4 **Pluripotency of riPS cells.** Expression of the pluripotency markers Oct4, Nanog, and SSEA-1 in the primary riPS cell clones IVB3 (A) and IVF3 (B) (passage 13), shown by immuncytochemical staining with respective antibodies. (C) Alkaline phosphatase staining in the same two clones. (D) RT-PCR analysis of pluripotency (Nanog), ectoderm (NCAM), mesoderm (FLK and AFP), and endoderm (Sox17 and GATA4) lineage marker expression during the time course of differentiation of the same riPS cell clones using embryoid body (EB) protocols. Cells were harvested at the indicated days after the EB formation (D0-D10). Nat1 served as an endogenous mRNA control. NC- negative control.
(DOC)

Table S1 **LIF and inhibition of GSK3 and MEK1/ERK signaling are essential for the riPS cell self-renewal.** Cells were seeded at a density of 50 cells/cm^2^ in a 6-well plate and cultured in different conditions for 5 days. Total number of surviving colonies, number of undifferentiated (AP-positive) colonies and number of morphologically differentiated colonies with partial AP-staining, which appeared specifically in the absence of PD0325901 (see Figure S2C, D) were counted. *p<0.01, **p<0.001 vs CHIR99021 + PD0325901 + LIF condition (one way ANOVA with Bonferroni's post test, N = 3 per condition. Similar results were obtained in 2 independent experiments.
(DOC)

Table S2 **Summary of subclones derived from riPS cell clone IIIB9 after Cre-mediated excision of proviruses and co-electroporation with** ***p2A2Btk-TKiresPuro*** **cassette.**
(DOC)

Table S3 **List of primer pairs used in this study.**
(DOC)

Table S4 **Real time PCR analysis of E18 chimeric embryos.** RiPS cells, harboring *2A2Btk-TKiresPuro* cassette in their genome (clone H5) were injected into 8-cell rat embryos. DNA isolated from fetuses at E18 was analyzed by real time PCR using a primer pair specific for the *2A2Btk-TKiresPuro* cassette (Table S3 and Figure S3B) normalized to the rat angiotensinogen (*AOGEN)* gene (this gene is present in both host embryo and incorporated riPS cells). H5 – riPS cell clone used for the injection, 12 – positive E18 fetus. T – tail, L – liver, H – head, K – kidney, P – placenta.
(DOC)
