# Supplementary material for: Derivation, Characterization, and Stable Transfection of Induced Pluripotent Stem Cells from Fischer344 Rats
Source: PLoS One. 2011 Nov 4;6(11):e27345. doi: 10.1371/journal.pone.0027345 (PMC3208629; doi:10.1371/journal.pone.0027345)
Supplement: Figure S1 — Lentivirus silencing in undifferentiated riPS cells. (A) riPS cell clone IIIB9 after 22 passages (p22) in culture. Notice round compact, morphologically undifferentiated colonies that have lost EGFP expression. (B) FACS for EGFP+ cells of riPS cell clone IIIB9 p22 (lower panel) and its subclone IIIB9-G3 derived following the excision of proviruses, including EGPF. The latter served as a negative control (upper panel). Similar results were obtained in 2 independent experiments. (C) Real time PCR analysis of sorted cells, showing that EGFP— riPS cells express high levels of Nanog, confirming the pluripotent state of this cell population. IIIB9: N = 3; EGFP— , EGFP+: N = 2. (D) The EGFP— fraction of riPS cells after FACS shows the onset of EGFP expression in some cells already after 1 day in culture. (E) EGFP— fraction cultured for further 4 days shows a substantial number of EGFP+ cells. Note, that most of Oct4+ cells do not express EGFP, whereas some of morphologically differentiated cells became green. (DOC) [file pone.0027345.s001.doc]

**Figure S1.** **Lentivirus silencing in undifferentiated riPS cells**. **(A)** riPS cell clone IIIB9 after 22 passages (p22) in culture. Notice round compact, morphologically undifferentiated colonies that have lost EGFP expression. (**B**) FACS for EGFP^+^ cells of riPS cell clone IIIB9 p22 (lower panel) and its subclone IIIB9-G3 derived following the excision of proviruses, including *EGPF*. The latter served as a negative control (upper panel). Similar results were obtained in 2 independent experiments. **(C)** Real time PCR analysis of sorted cells, showing that EGFP^—^ riPS cells express high levels of Nanog, confirming the pluripotent state of this cell population. IIIB9: N=3; EGFP^—^ , EGFP^+^: N=2. (**D**) The EGFP^—^ fraction of riPS cells after FACS shows the onset of EGFP expression in some cells already after 1 day in culture. **(E)** EGFP^—^ fraction cultured for further 4 days shows a substantial number of EGFP^+^ cells. Note, that most of Oct4^+^ cells do not express EGFP, whereas some of morphologically differentiated cells became green.
