# Supplementary material for: Derivation, Characterization, and Stable Transfection of Induced Pluripotent Stem Cells from Fischer344 Rats
Source: PLoS One. 2011 Nov 4;6(11):e27345. doi: 10.1371/journal.pone.0027345 (PMC3208629; doi:10.1371/journal.pone.0027345)
Supplement: Figure S2 — RiPS cell clonability assay. Representative images of alkaline phosphatase staining of riPS cells, seeded at a density of 500 cells per well of a 6-well plate and cultured for 5 days in the presence of LIF and 2i (A, B), or in the presence of LIF and CHIR99021 (C, D). 2.5x magnification (A, C). Examples of an undifferentiated colony (B) and of a morphologically differentiated colony with weak AP-staining (D) (20x and 10x magnification, respectively). Similar results were obtained in 2 independent experiments. (DOC) [file pone.0027345.s002.doc]

**Figure S2. RiPS cell clonability assay.** Representative images of alkaline phosphatase staining of riPS cells, seeded at a density of 500 cells per well of a 6-well plate and cultured for 5 days in the presence of LIF and 2i (A, B), or in the presence of LIF and CHIR99021 (C, D). 2.5x magnification (A, C). Examples of an undifferentiated colony (B) and of a morphologically differentiated colony with weak AP-staining (D) (20x and 10x magnification, respectively). Similar results were obtained in 2 independent experiments.

**Suppl. Figure 3. Plasmids and lentiviruses.** (A) Map of the lentiviral vectors used in this study. Oct4, Sox2, Klf4, and cMyc cDNAs were cloned in place of the EGFP cDNA of the LVTHM vector, which is described in greater details in elsewhere (Wiznerowicz and Trono, 2003, *J. Virol* 77:8957-61). During reverse transcription, the U3 region of the 5’ LTR is synthesized by using its 3’ homologue as a template, which results in a duplication of LoxP site in the provirus integrated in the genome of transduced cells. The part of LV DNA between the loxP sites is subject to Cre-mediated excision. LTR, cPPT, and WPRE are lentiviral elements required for its integration and expression. (B) Scheme of the *2A2Btk-TKiresPuro* cassette (see text for abbreviations). Arrows indicate primers which were used to detect the cassette in the riPS transfected clones. The same primer pair was employed for the evaluation of % chimerism after injection of riPS H5 cells into rat preimplantation embryos (Figure 2E and Suppl. Table 4).


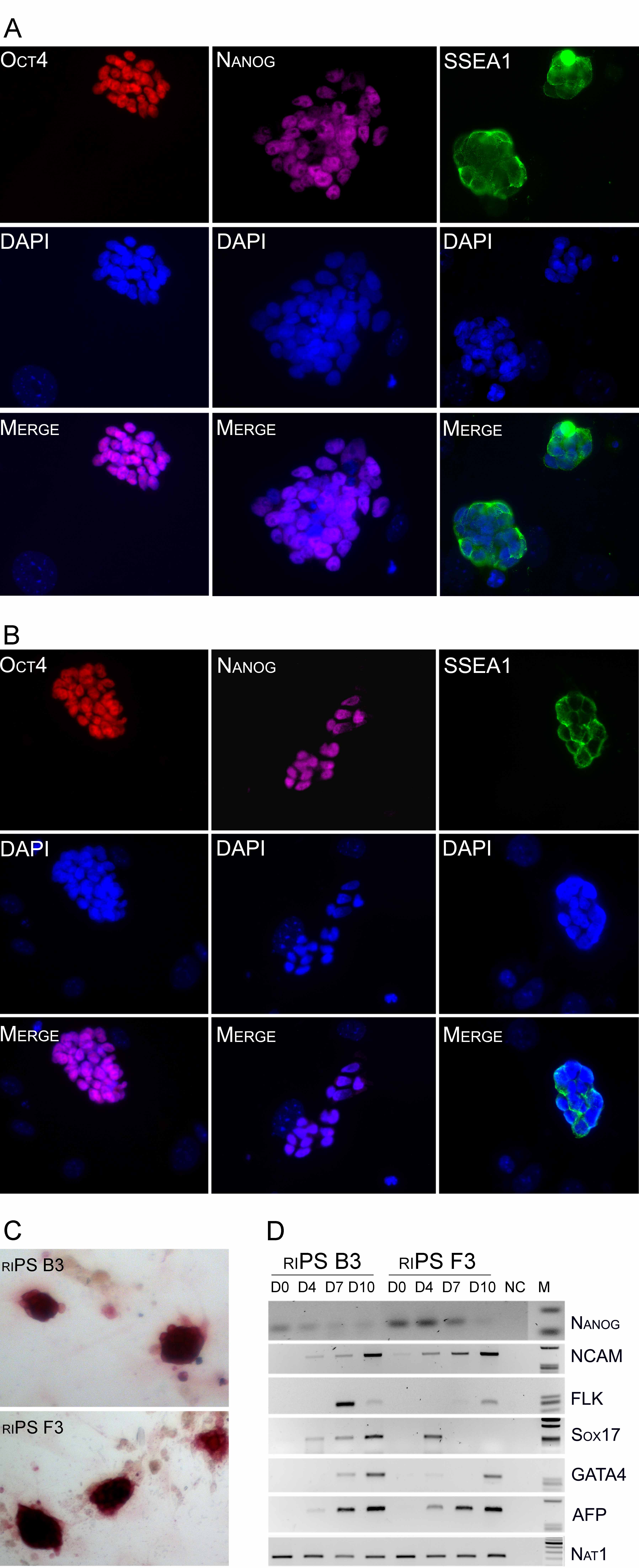


**Suppl. Figure 4.** Expression of the pluripotency markers Oct4, Nanog, and SSEA-1 in the primary riPS cell clones IVB3 (A) and IVF3 (B) (passage 13), shown by immuncytochemical staining with respective antibodies. (С) Alkaline phosphatase staining in the same two clones. (D) RT-PCR analysis of pluripotency (Nanog), ectoderm (NCAM), mesoderm (FLK and AFP), and endoderm (Sox17 and GATA4) lineage marker expression during the time course of differentiation of the same riPS cell clones using embryoid body (EB) protocols. Cells were harvested at the indicated days after the EB formation (D0-D10). Nat1 served as an endogenous mRNA control. NC- negative control.
