# Supplementary material for: Derivation, Characterization, and Stable Transfection of Induced Pluripotent Stem Cells from Fischer344 Rats
Source: PLoS One. 2011 Nov 4;6(11):e27345. doi: 10.1371/journal.pone.0027345 (PMC3208629; doi:10.1371/journal.pone.0027345)
Supplement: Figure S3 — Plasmids and lentiviruses. (A) Map of the lentiviral vectors used in this study. Oct4, Sox2, Klf4, and cMyc cDNAs were cloned in place of the EGFP cDNA of the LVTHM vector, which is described in greater details in elsewhere (Wiznerowicz and Trono, 2003, J. Virol 77:8957-61). During reverse transcription, the U3 region of the 5′ LTR is synthesized by using its 3′ homologue as a template, which results in a duplication of LoxP site in the provirus integrated in the genome of transduced cells. The part of LV DNA between the loxP sites is subject to Cre-mediated excision. LTR, cPPT, and WPRE are lentiviral elements required for its integration and expression. (B) Scheme of the 2A2Btk-TKiresPuro cassette (see text for abbreviations). Arrows indicate primers which were used to detect the cassette in the riPS cell transfected clones. The same primer pair was employed for the evaluation of % chimerism after injection of riPS H5 cells into rat preimplantation embryos (Figure 2E and Table S4). (DOC) [file pone.0027345.s003.doc]

**Figure S3. Plasmids and lentiviruses.** (A) Map of the lentiviral vectors used in this study. Oct4, Sox2, Klf4, and cMyc cDNAs were cloned in place of the EGFP cDNA of the LVTHM vector, which is described in greater details in elsewhere (Wiznerowicz and Trono, 2003, *J. Virol* 77:8957-61). During reverse transcription, the U3 region of the 5’ LTR is synthesized by using its 3’ homologue as a template, which results in a duplication of LoxP site in the provirus integrated in the genome of transduced cells. The part of LV DNA between the loxP sites is subject to Cre-mediated excision. LTR, cPPT, and WPRE are lentiviral elements required for its integration and expression. (B) Scheme of the *2A2Btk-TKiresPuro* cassette (see text for abbreviations). Arrows indicate primers which were used to detect the cassette in the riPS cell transfected clones. The same primer pair was employed for the evaluation of % chimerism after injection of riPS H5 cells into rat preimplantation embryos (Figure 2E and Table S4).
