# Supplementary material for: Derivation, Characterization, and Stable Transfection of Induced Pluripotent Stem Cells from Fischer344 Rats
Source: PLoS One. 2011 Nov 4;6(11):e27345. doi: 10.1371/journal.pone.0027345 (PMC3208629; doi:10.1371/journal.pone.0027345)
Supplement: Figure S4 — Pluripotency of riPS cells. Expression of the pluripotency markers Oct4, Nanog, and SSEA-1 in the primary riPS cell clones IVB3 (A) and IVF3 (B) (passage 13), shown by immuncytochemical staining with respective antibodies. (C) Alkaline phosphatase staining in the same two clones. (D) RT-PCR analysis of pluripotency (Nanog), ectoderm (NCAM), mesoderm (FLK and AFP), and endoderm (Sox17 and GATA4) lineage marker expression during the time course of differentiation of the same riPS cell clones using embryoid body (EB) protocols. Cells were harvested at the indicated days after the EB formation (D0-D10). Nat1 served as an endogenous mRNA control. NC- negative control. (DOC) [file pone.0027345.s004.doc]

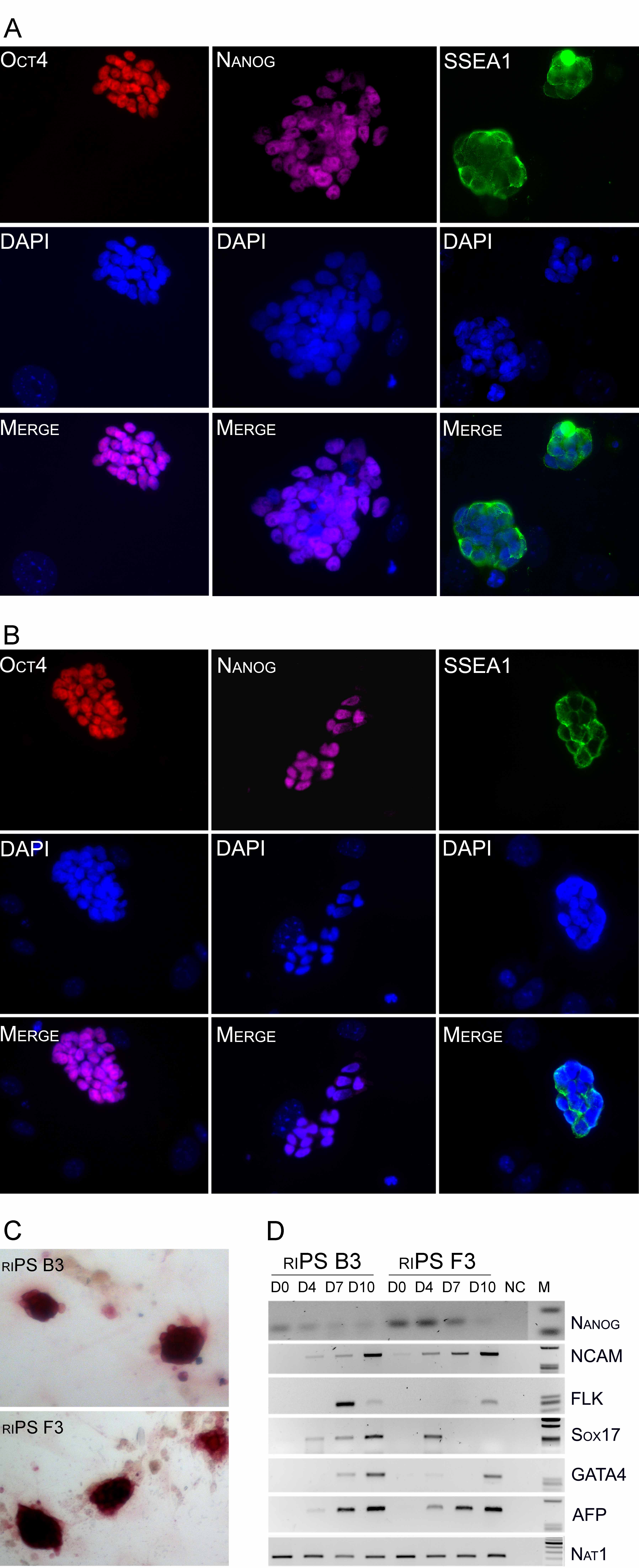


**Figure S4.** **Pluripotency of riPS cells.** Expression of the pluripotency markers Oct4, Nanog, and SSEA-1 in the primary riPS cell clones IVB3 (A) and IVF3 (B) (passage 13), shown by immuncytochemical staining with respective antibodies. (С) Alkaline phosphatase staining in the same two clones. (D) RT-PCR analysis of pluripotency (Nanog), ectoderm (NCAM), mesoderm (FLK and AFP), and endoderm (Sox17 and GATA4) lineage marker expression during the time course of differentiation of the same riPS cell clones using embryoid body (EB) protocols. Cells were harvested at the indicated days after the EB formation (D0-D10). Nat1 served as an endogenous mRNA control. NC- negative control.
