# Supplementary material for: Derivation, Characterization, and Stable Transfection of Induced Pluripotent Stem Cells from Fischer344 Rats
Source: PLoS One. 2011 Nov 4;6(11):e27345. doi: 10.1371/journal.pone.0027345 (PMC3208629; doi:10.1371/journal.pone.0027345)
Supplement: Table S1 — LIF and inhibition of GSK3 and MEK1/ERK signaling are essential for the riPS cell self-renewal. Cells were seeded at a density of 50 cells/cm2 in a 6-well plate and cultured in different conditions for 5 days. Total number of surviving colonies, number of undifferentiated (AP-positive) colonies and number of morphologically differentiated colonies with partial AP-staining, which appeared specifically in the absence of PD0325901 (see Figure S2C, D) were counted. *p<0.01, **p<0.001 vs CHIR99021 + PD0325901 + LIF condition (one way ANOVA with Bonferroni's post test, N = 3 per condition. Similar results were obtained in 2 independent experiments. (DOC) [file pone.0027345.s005.doc]

**Table S1. LIF and inhibition of GSK3 and MEK1/ERK signaling are essential for the riPS cell self-renewal.**

| **Condition** | **total N of surviving colonies** | **N of AP-positive colonies** | **N of partially**  **AP-positive colonies*** |
| --- | --- | --- | --- |
| CHIR99021 + PD0325901 + LIF | 28.3±7.1 | 27.0±8.5 |  |
| CHIR99021 + LIF | 11.3±4.2 * | 4.3±3.2 ** | 5.3±2.1 |
| PD0325901 + LIF | 0 ** | 0 ** |  |
| CHIR99021 | 9.0±1.0 ** | 0 ** | 6.3±2.1 |
| PD0325901 | 0 ** | 0 ** |  |

Cells were seeded at a density of 50 cells / cm^2^ in a 6-well plate and cultured in different conditions for 5 days. Total number of surviving colonies, number of undifferentiated (AP-positive) colonies and number of morphologically differentiated colonies with partial AP-staining, which appeared specifically in the absence of PD0325901 (see Figure S2C, D) were counted. *p<0.01, **p<0.001 vs CHIR99021 + PD0325901 + LIF condition (one way ANOVA with Bonferroni’s post test, N=3 per condition. Similar results were obtained in 2 independent experiments.
