# Supplementary material for: Derivation, Characterization, and Stable Transfection of Induced Pluripotent Stem Cells from Fischer344 Rats
Source: PLoS One. 2011 Nov 4;6(11):e27345. doi: 10.1371/journal.pone.0027345 (PMC3208629; doi:10.1371/journal.pone.0027345)
Supplement: Table S2 — Summary of subclones derived from riPS cell clone IIIB9 after Cre-mediated excision of proviruses and co-electroporation with p2A2Btk-TKiresPuro cassette. (DOC) [file pone.0027345.s006.doc]

**Table S2. Summary of subclones derived from riPS cell clone IIIB9 after Cre-mediated excision of proviruses and co-electroporation with *p2A2Btk-TKiresPuro* cassette.**

| **subclone** | **lenti-*Oct4*** | **lenti-*Sox2*** | **lenti-*Klf4*** | **lenti-*cMyc*** | **lenti-*EGFP*** | ***p2A2Btk-TKiresPuro*** |
| --- | --- | --- | --- | --- | --- | --- |
| **H5** | **-** | **-** | **-** | **-** | **-** | **+** |
| **G3** | **+** | **-** | **-** | **-** | **-** | **+** |
| **G4** | **-** | **-** | **-** | **-** | **-** | **-** |
