# Supplementary material for: Derivation, Characterization, and Stable Transfection of Induced Pluripotent Stem Cells from Fischer344 Rats
Source: PLoS One. 2011 Nov 4;6(11):e27345. doi: 10.1371/journal.pone.0027345 (PMC3208629; doi:10.1371/journal.pone.0027345)
Supplement: Table S3 — List of primer pairs used in this study. (DOC) [file pone.0027345.s007.doc]

**Table S3. List of primer pairs used in this study.**

| **Target gene** | **Species** |  | **Primer sequence** | **Application** |
| --- | --- | --- | --- | --- |
| Oct4 | Lenti | Fw primer (5’->3’) | GCCAAGGCAAGGGAGGTAGAC | Genomic DNA |
|  |  | Rev primer (5’->3’) | GCCATACGGGAAGCAATAGCA |  |
| Sox2 | Lenti | Fw primer (5’->3’) | CCAGCTCGCAGACCTACATGA | Genomic DNA |
|  |  | Rev primer (5’->3’) | GCCATACGGGAAGCAATAGCA |  |
| Klf4 | Lenti | Fw primer (5’->3’) | TGACCAGGCACTACCGCAAAC | Genomic DNA |
|  |  | Rev primer (5’->3’) | GCCATACGGGAAGCAATAGCA |  |
| cMyc | Lenti | Fw primer (5’->3’) | AGAGGCGGACACACAACGTC | Genomic DNA |
|  |  | Rev primer (5’->3’) | GCCATACGGGAAGCAATAGCA |  |
| EGFP | Lenti | Fw primer (5’->3’) | GCAAGCTGACCCTGAAGTTCATC | Genomic DNA |
|  |  | Rev primer (5’->3’) | GCCATACGGGAAGCAATAGCA |  |
| Sry | mouse & | Fw primer (5’->3’) | GACTATCGATTGTCTAGAGAGCATGGA | Genomic DNA |
|  | rat | Rev primer (5’->3’) | GACTGTCGACTAACTCCTCTGNGGCACT |  |
| TK | p2A2Btk- | Fw primer (5’->3’) | CCGCCCTCCTGTGCTACC | Genomic DNA |
|  | TKiresPuro cassette | Rev primer (5’->3’) | GCAGATACCGCACCGTATTGG |  |
| AOGEN | rat | Fw primer (5’->3’) | GAGTGAGGCAAGAGGAGGTGTAG | Genomic DNA |
|  |  | Rev primer (5’->3’) | CCCAAGCTCTCAACAAATGGC |  |
| Nanog | mouse & | Fw primer (5’->3’) | CTGAACCTGAGCTATAAGCAG | RT-PCR & |
|  | rat | Rev primer (5’->3’) | ACCA T/C TGGTTTTTCTGCCACC | qPCR |
| Nat1 | mouse & | Fw primer (5’->3’) | ATTCTTCGTTGTCAAGCCGCCAAAGTGGAG | RT-PCR & |
|  | rat | Rev primer (5’->3’) | AGTTGTTTGCTGCGGAGTTGTCATCTCGTC | qPCR |
| Gata4 | rat | Fw primer (5’->3’) | GCATCCATTTCCACCTCTT | RT-PCR |
|  |  | Rev primer (5’->3’) | TCCATCACCCTTGTCCTTT |  |
| AFP | rat | Fw primer (5’->3’) | GTCCCACCCTTCCACTTT | RT-PCR |
|  |  | Rev primer (5’->3’) | CCATCCTGTAGGCACTCC |  |
| FLK | rat | Fw primer (5’->3’) | ATACACCTGCACAGCGTACAG | RT-PCR |
|  |  | Rev primer (5’->3’) | TCCCGCATCTCTTTCACTCAC |  |
| Sox17 | rat | Fw primer (5’->3’) | AGGAGAGGTGGTGGCGAGTAG | RT-PCR |
|  |  | Rev primer (5’->3’) | GTTGGGATGGTCCTGCATGTG |  |
| Nestin | rat | Fw primer (5’->3’) | AGCCATTGTGGTCTACTGA | RT-PCR |
|  |  | Rev primer (5’->3’) | TGCAACTCTGCCTTATCC |  |
| NCAM | rat | Fw primer (5’->3’) | TGCTCAAGTCCCTAGACTGGAACG | RT-PCR |
|  |  | Rev primer (5’->3’) | CTTCTCGGGCTCTGTCAGTGGTGTGG |  |
| TBP | mouse & | Fw primer (5’->3’) | CCCTATCACTCCTGCCACACC | RT-PCR |
|  | rat | Rev primer (5’->3’) | CGAAGTGCAATGGTCTTTAGGTC |  |
