# Supplementary material for: Derivation, Characterization, and Stable Transfection of Induced Pluripotent Stem Cells from Fischer344 Rats
Source: PLoS One. 2011 Nov 4;6(11):e27345. doi: 10.1371/journal.pone.0027345 (PMC3208629; doi:10.1371/journal.pone.0027345)
Supplement: Table S4 — Real time PCR analysis of E18 chimeric embryos. RiPS cells, harboring 2A2Btk-TKiresPuro cassette in their genome (clone H5) were injected into 8-cell rat embryos. DNA isolated from fetuses at E18 was analyzed by real time PCR using a primer pair specific for the 2A2Btk-TKiresPuro cassette (Table S3 and Figure S3B) normalized to the rat angiotensinogen (AOGEN) gene (this gene is present in both host embryo and incorporated riPS cells). H5 – riPS cell clone used for the injection, 12 – positive E18 fetus. T – tail, L – liver, H – head, K – kidney, P – placenta. (DOC) [file pone.0027345.s008.doc]

**Table S4. Real time PCR analysis of E18 chimeric embryos.**

| **Tissue** | **% chimerism** |
| --- | --- |
| 12T | 17 % |
| 12H | 23 % |
| 12Li | 38 % |
| 12K | 22 % |
| 12P | nd |
| riPS H5 | 100 % |

RiPS cells, harboring *2A2Btk-TKiresPuro* cassette in their genome (clone H5) were injected into 8-cell rat embryos. DNA isolated from fetuses at E18 was analyzed by real time PCR using a primer pair specific for the *2A2Btk-TKiresPuro* cassette (Table S3 and Figure S3B) normalized to the rat angiotensinogen (*AOGEN)* gene (this gene is present in both host embryo and incorporated riPS cells). H5 – riPS cell clone used for the injection, 12 – positive E18 fetus. T – tail, L – liver, H – head, K – kidney, P – placenta.
